# Determining factors of the blood feeding behaviour of mosquito vectors of West Nile and Rift Valley fever viruses in Madagascar

**DOI:** 10.1038/s41598-026-46448-3

**Published:** 2026-04-28

**Authors:** Luciano Michaël Tantely, Hélène Guis, Manou Rominah Raharinirina, Fidelis Maminirina Ambinintsoa, Sylviane Miharisoa, Mandaniaina Radotiana Andriamiarimanana, Ignace Rakotoarivony, Miora Felaniaina Andrianandrasana, Thiery Nirina Nepomichene, Zara Nomentsoa Razafiarimanga, Diego Ayala, Claire Garros, Catherine Cêtre-Sossah, Jean-Michel Héraud, Annelise Tran, Thomas Walker, Romain Girod

**Affiliations:** 1 Medical Entomology Unit, Institut Pasteur de Madagascar, Antananarivo, Madagascar; 2 CIRAD, UMR ASTRE, Antananarivo, Madagascar; 3 Epidemiology and Clinical Research Unit, Institut Pasteur de Madagascar, Antananarivo, Madagascar; 4 CIRAD, UMR ASTRE, Montpellier, France; 5 CIRAD, INRAE, ASTRE, Université de Montpellier, Montpellier, France; 6 Department of Biochemistry, University of Antananarivo, Antananarivo, Madagascar; 7 UMR 224, MIVEGEC/BEES, IRD, Montpellier, France; 8 CIRAD, UMR ASTRE, Sainte-Clotilde, La Réunion, France; 9 Virology Unit, Institut Pasteur de Madagascar, Antananarivo, Madagascar; 10 CIRAD, UMR TETIS, Montpellier, F-34398 France; 11 TETIS, Université de Montpellier, CIRAD, INRAE, AgroParisTech, Montpellier, France; 12 School of Life Sciences, University of Warwick, Coventry, UK

**Keywords:** Mosquito vectors, Arboviruses, Blood feeding patterns, Climatic and environmental drivers, Multiple-site model, Madagascar, Ecology, Ecology, Zoology

## Abstract

In Madagascar, the health burdens caused by West Nile virus (WNV) and Rift Valley Fever virus (RVFV) vary across regions, likely resulting from the variations of their vector capacity. This study aimed to assess the degree of vector-hots contact and to develop statistical models to identify factors influencing host preferences. Bimonthly mosquito collections were performed in 25 sites of Madagascar from February to October 2019. Engorged mosquitoes were collected from indoors and outdoors resting locations within each site. Blood meal identification was performed using host-specific PCRs. Generalised linear models were developed to explain host preferences of the two most abundant species. The origin of the blood meal of 1,177 engorged females belonging to ten species was analysed. Human and cattle were the dominant vertebrate hosts identified (respectively 44.86% and 44.52%), and pigs were rarely detected (< 1%). Models were developed for *Culex antennatus* and *Culex quinquefasciatus*. For these two species, host preference was driven by climatic and environmental factors, type of habitat, indoor/outdoor location, and number of available vertebrate hosts. This study provides an essential step towards an evidence-based determination of arbovirus transmission risk and suggests to explore the use of other tools to supplement indoor vector control in Madagascar.

**Supplementary Information:**

The online version contains supplementary material available at 10.1038/s41598-026-46448-3.

## Introduction

The dissemination of mosquito-borne viruses relies on the bite of mosquito on a vertebrate host to obtain a blood meal^1^. The presence, abundance and diversity of vertebrate hosts have an impact on mosquito feeding behaviour^2,3^. This is the case for generalist feeders, but also for specialist feeders (anthropophilic, ornithophilic or zoophilic). Mosquito feeding behaviour is influenced by intrinsic determinants of mosquito host preferences, and by numerous external factors linked to the host, climatic and environmental factors^4,5^.

Host-related factors impacting mosquito feeding behaviour include abundance, host species, host relative body heat, body mass, color and gender^6^. Amount of carbon dioxide and (S)-lactic released, specific skin microbial profile, blood quality, defensive behaviour against insect bites, infection by pathogens are also reported to impact mosquito feeding behaviour^4^. Climatic factors such as relative humidity and precipitation are important factors^3,4^. Specifically, in Madagascar, the effects of both intrinsic and extrinsic factors on mosquito feeding behaviour have only been mentioned in descriptive studies^7,8^ so further studies are warranted to quantify these associations.

Currently, only 55 out of the 237 mosquito species recorded in Madagascar are known to be specialist feeders or generalist feeders^7,9^. Among these 55 species, some are considered as potential, candidates or major vectors of human pathogens such as malaria parasites or arboviruses^7^.

Twelve arboviruses have been confirmed to occur in Madagascar^10–12^. Among them, RVFV and WNV are the most abundant, most widespread and best studied^10,13,14^. They are both zoonotic^12,15^, with the former displaying an epizootic and epidemic circulations and the latter an enzootic silent but widespread circulation^10,11,14,16^. Cattle, sheep and goats are the primary vertebrate hosts for RVFV while wild and domestic birds are the primary vertebrate hosts for WNV^17^. The burden of WNV and RVFV vary across different regions within Madagascar^13,14,18–20^. This is likely due to the difference in mosquito species composition and difference in mosquito abundance between regions^10^, host abundance^13^, trade routes^19^ and the level of vector-host (humans and/or other animals) contacts.

However, In Madagascar, numerous data on the feeding preferences of mosquito vectors of WNV and RVFV are limited in time and space and does not allow to evaluate feeding variations among populations and species^10,21–23^.

This large-scale study aimed at: (i) assessing the degree of contact between RVFV and WNV mosquito vector species and their host in Madagascar, (ii) providing the first insights on the influence of climatic and environmental factors on the variability in mosquito feeding behaviours, and (iii) developing statistical models to identify factors influencing the presence/absence of blood in human, cattle, bird, and small ruminant (sheep and goat combined) (SRu) in blood-fed mosquitoes, serving as a proxy for human and household exposure to mosquito bites. A better understanding of mosquito feeding behaviour could contribute to better target vector control strategies (by guiding the choice between sprays, mosquito nets or larval control) to specific species that are most likely to be responsible for the widespread transmission of arboviruses.

## Methods

### Mosquito collection locations and periods

**Fig. 1:**
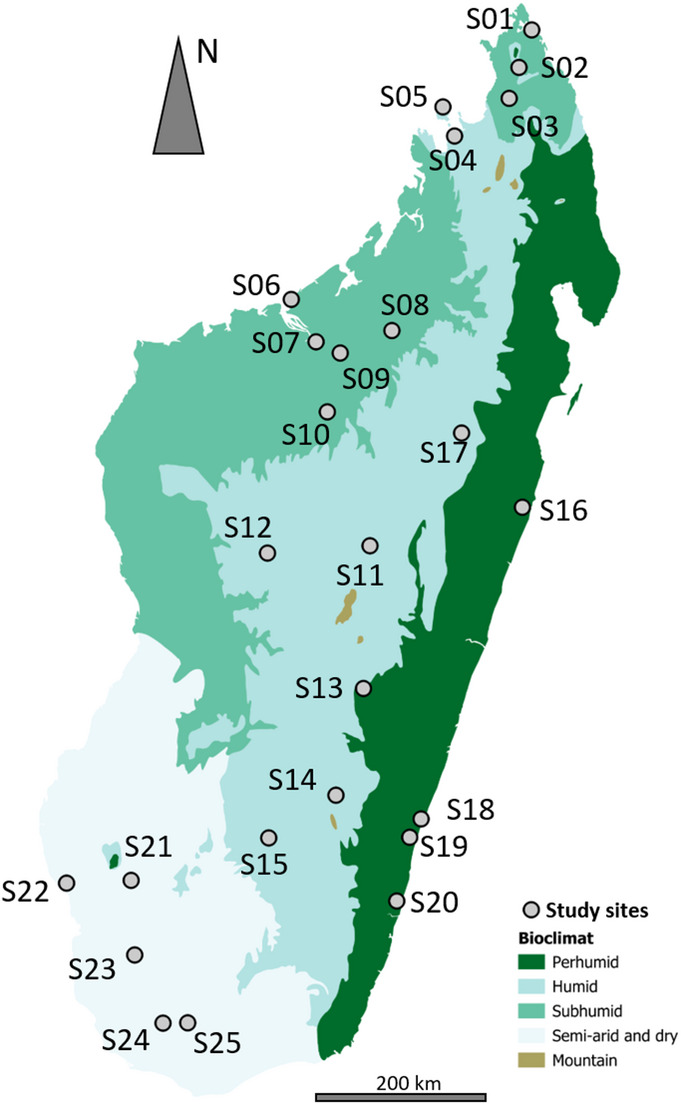
Map showing the five climatic zones and the locations of the 25 study sites (grey circle, numbered S01 to S25) investigated from February to December 2019. This map was created using QGIS Version 3.38.1 and using Microsoft Powerpoint (2019).

This study was conducted as part of a larger entomological monitoring survey undertaken at a national scale and over two years^24^. This larger entomological monitoring survey included four types of mosquito collection methods: the use of light traps, human-baited double net traps, and mosquito collection from indoor pyrethrum spray catches (PSCs) and Muirhead-Thomson pit traps (MTPTs). In this present study, we present only results from engorged females obtained from PSCs and MTPTs. Collected mosquitoes were obtained from 25 villages, located in 24 districts distributed across the entire country and covering the six climatic zones of Madagascar defined by Brunhes et al^9^. (Fig. 1, Supplementary Table S1). They were collected in February, April, June, August and October 2019.

### Mosquito collection methods

**Fig. 2:**
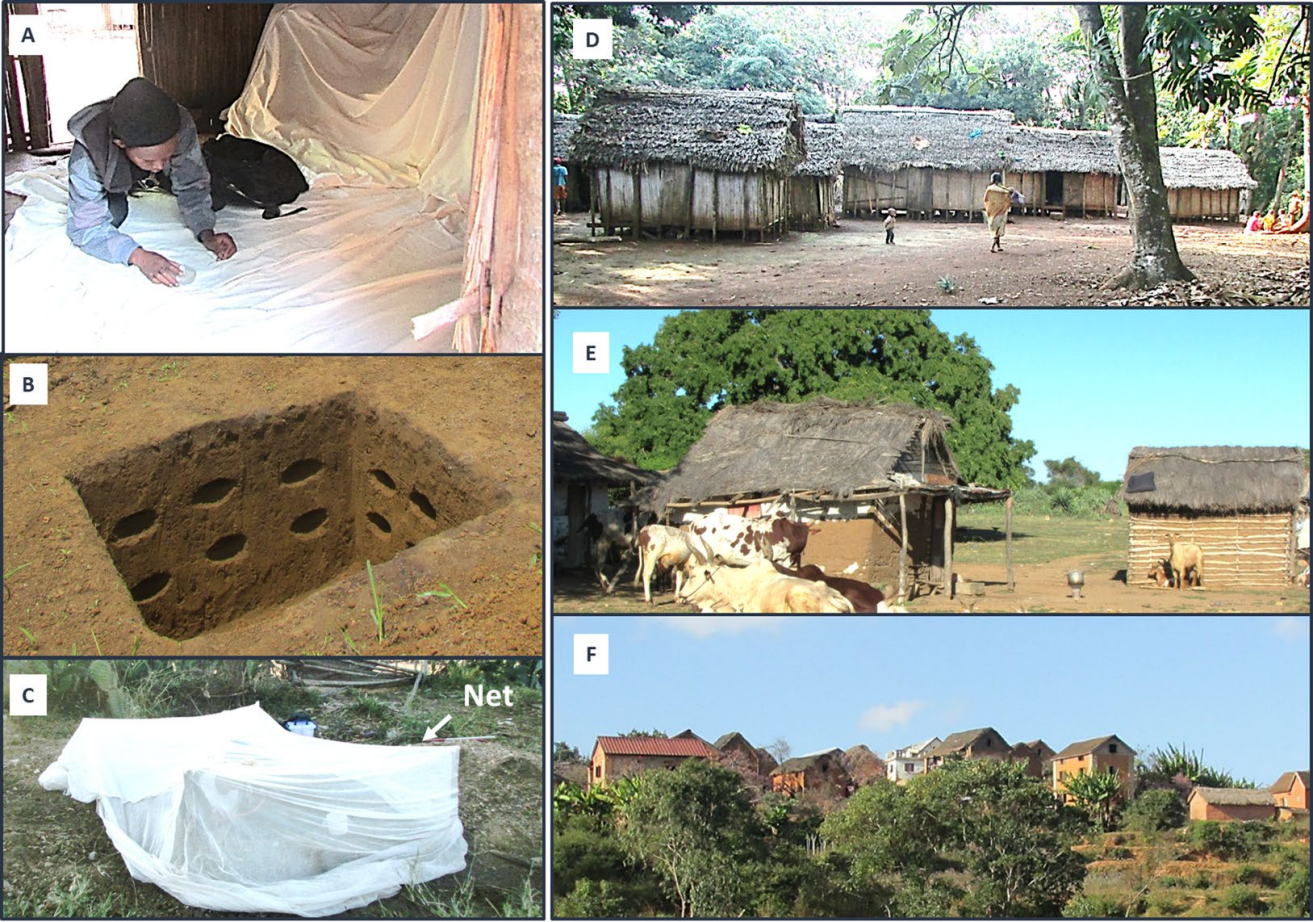
Mosquito collection settings and methods. **A** Michaël Luciano Tantely performing indoor mosquito collection, **B** Muirhead Thomson pit trap before mosquito collection; **C** Michaël Luciano Tantely performing mosquito collection in the improved Muirhead Thomson pit trap (the net was used to cover the MTPT to prevent mosquitoes from escaping during collection); **D–F**: Typical housing in Madagascar (**C** = Ravinala made wall and roofs; **D** = mud walls and roofs made of palms leaves sometimes combined with corrugated metal sheets; **E** = brick walls with thatched, metal or tiled roofs).

Endophilic engorged mosquitoes were collected indoor, using commercial insecticide aerosol sprays applied to the inner walls and roofs (indoor pyrethrum spray catches, PSC) (Fig. 2A) and exophilic engorged mosquitoes using improved Muirhead-Thomson pit traps (MTPT) (Fig. 2B and C).

The PSC consists of indoor spraying of a commercial insecticide aerosol spray (Ramp Aik Extra^®^) to collect mosquitoes resting inside houses. It was performed in three houses per site and located: one near a cattle park, one in the centre of the study site (and not close to a cattle park) and one near a water source (rice field and large permanent water body). Firstly, all windows and doors were closed and white sheets were laid on the floor of the room. Then, insecticide was sprayed for 45–60 s. After 10 min of insecticide exposure, the immobilized mosquitoes were recovered from the white sheet using forceps. Mosquitoes were collected once per day (between 07:00 and 08:00 every morning) during two consecutive days.

MTPTs are cube-shaped pits with small cavities in the walls. To collect the exophilic engorged mosquitoes, six MTPTs were dug from each of the three houses where PSCs were performed. The opening of each MTPT was recovered with nets placed over support stakes just before the start of the mosquito collection (at around 07:30) to prevent resting mosquitoes from escaping. Resting mosquitoes were sampled in each pit using a mouth aspirator. As for PSC, mosquitoes were collected in MTPT once per day during two consecutive days. For the MTPT, the total sampling effort was 6 MTPT sampled × 2 days × 25 sites × 5 field work session, corresponding to a total of 1500 MTPTs sampled. For PSC, the total sampling effort was 3 houses sampled × 2 days × 25 sites × 5 field work session, corresponding to a total of 750 houses sampled.

### Mosquito identification and processing

In the field, adult mosquitoes were killed with chloroform vapor and morphologically identified using the unpublished Fontenille keys. After identification, mosquitoes were counted, pooled according to species, sex, blood feeding status (engorged or non-engorged), date of capture, type of traps, study site and location of traps. Mosquito pools were stored in liquid nitrogen in the field before being stored in the laboratory at −80 °C pending further analysis.

### DNA extraction and amplification for blood meal source identification

Engorged females of species reported to be associated with RVFV and WNV transmission^25,26^ were used for blood meals identification. DNA from whole body of engorged mosquitoes was extracted individually using Qiagen DNeasy blood and tissue kit (Qiagen, Germany). Extracted DNA was eluted in 200 µL of AE elution buffer according to the manufacturer’s instructions and stored at −20 °C until use. Host primers (targeting cytochrome b polymorphism of humans, cattle, sheep, goats, Galliformes and Passeriformes)^27–29^ were used (Table 1), as they are involved in the transmission cycles of WNV and RVFV. Primers to detect pig^27^ blood was used as they were abundant in our study sites.

**Table 1:** Primer sets used for the identification of blood meal origin in engorged female mosquitoes.

Target	Reverse: 5’−3’ sequence	Forward 5’−3’ sequence	Product sizes	References
Cattle	AGTGGGYGRAATATT ATGC	TTATCATCATAGCAA TTGCC	400	^29^
Sheep	GGCGTGAATAGTACT AGTAGCATGAGGATGA	TGAGGACAAATATCA TTYTGAGGRGC	336	^27^
Goat	TTAGAACAAGAATTA GTAGCATGGCG	TGAGGACAAATATCA TTYTGAGGRGC	313	^27^
Human	AGTGGGYGRAATATT ATGC	CTCGGCTTACTTCTC TTCC	272	^29^
Passeriformes	AAYGGGTGTTCKACT GGTTGGCT	RGCMCTRGCHGCCTC MRTCCTAG	165	^28^
Galliformes	GTCCGATGTGAAGGA AGATACAGATGAAGAAGAA	ATTTCGGCTCCCTAT TAGCAG	210	^28^
Pig	TCTGATGTGTAATGT ATTGCTAAGAAC	GACCAATGATATGAA AAACCATCGTTGT	219	^27^

PCR reactions were performed using GoTaq Hot Start Green Master Mix (Promega, Madison, Wisconsin, USA) 1X final concentration, forward and reverse primers with a 1µM final concentration, 2µL of 1/10 diluted DNA in a final reaction volume of 25 µL. PCR amplifications were done using two types of thermocyclers depending on availability: Applied Biosystems Veriti 96-well thermal cycler (Applied Biosystems, Foster City, CA) or Thermo Scientific Arktik Thermal Cycler (Thermo Fisher Scientific Oy). PCR products were visualized on 2% agarose gels using Bio-Rad scanner (Bio‐Rad Universal Hood II).

### Host, environmental and climatic data

The number of cattle, chicken, SRu (number of sheep and goat combined) and pig per 10 km² was determined using the 2020 gridded livestock of the world map (https://dataverse.harvard.edu/dataverse/glw). The number of inhabitants per district was obtained from The Humanitarian Data Exchange^30^. Daily records of meteorological parameters (wind speed, precipitation, temperature and relative humidity [RH]) from January to December 2019 were obtained from NASA Langley Research Centre (LaRC)^31^. Rescaled (initial values divided by 100) mean daily values of Normalized Difference Vegetation Index (NDVI) and Normalized Difference Water Index (NDWI) were downloaded from the most recent Sentinel-2 image (originally at 10 m spatial resolution and 5-day frequency) of Sen2Extract web application^32^ before the mosquito collection date. Rescaled average values of NDVI and NDWI obtained during the week prior to sampling were then computed. The insecticide treated nets (ITN) use-to-access ratio (percentage of people with access to an ITN who actually slept under one the previous night) were from the United States President’s Malaria Initiative report^33^. The variables related to the environment were based on field observation: biotope (near cattle park, near water body and in the village far of water body and free of cattle park), and the type of house [Ravinala (Fig. 2D), mud-made (Fig. 2E) and brick-made (Fig. 2F)].

### Statistical analyses

Data analyses were performed in R version 4.2.2^34^. Stacked bar plots with the ggplot2 packages were created to visualize the percentage and numbers of mosquito species with simple and mixed blood meals combined. To show the detailed types of single and mixed blood meals found in tested engorged females, an interactive chord diagram was created using the chorddiag package^35^.

Cattle, sheep, goat, Galliformes, Passeriformes and pig blood indices (BIs) were calculated using a similar method to determine the HBI^36^. To compare the BIs among mosquito species and among study sites, the chi-square test of independence was performed using the “chisq.test” function Pearson residuals were visualized using the corrplot package^37^. Waffle plots showing the number and distribution of engorged females tested were created using the ggplot238 and waffle R packages^39^. The “fviz_pca_biplot()” function from the factoextra package^40^ was used to visualize the relationships between blood index and indoor/outdoor resting habitat and between the three trap locations.

To identify the drivers of presence/absence of human, cattle, bird and SRu blood in mosquito females, a binomial logistic classification model (binomial response with logit link) was developed for each of the two most abundant mosquito species. Univariable models followed by multivariable models were developed to explain the presence/absence of each given blood. The outcome of the model, which is the presence/absence of blood of a given host in engorged females of the two most abundant species, was classified as a binary variable: y = 1 if the individual had taken a blood meal on that host (i.e. tested positive by PCR for that host) and y = 0 if not (PCR-negative result for that host).

In the first step, for each mosquito species, an univariable model tested the association of the outcome with 30 explanatory variables (tested one by one) by applying a generalized linear model. These 30 explanatory variables including trap location (near cattle park, near water body and in the village-far of water body and free of cattle), resting habitat (indoor by PSC *versus* outdoor by MTPT), domain (southern, northern, western, central and eastern domains), four variables concerning the type of house (Ravinala-made/non-Ravinala-made, brick-made/non-brick-made, mud-made/non-mud-made, all type of house), seasons (dry or wet). human and animal densities (5 variables), 13 climatic variables (which included wind speed on the day of the trapping (1 variable), as well as precipitation (4 variables), temperature (4 variables) and relative humidity (4 variables), environmental variables (NDVI and NDWI the week prior the sampling), built-up surfaces area per fokontany (smallest administrative division in Madagascar, functioning like a neighbourhood or local village, forming part of a larger municipality), and ITNs use-to-access ratio were included as continuous variables.

For this purpose, the variables of average temperature, average relative humidity and cumulative precipitation were calculated for the following four lag periods: day 1 (sampling day), 1–2 (sampling days and first day before the sampling period combined), 2–3 (first and second days before the sampling period combined) and 1–3 (sampling days, first and second days before the sampling period combined).

In the second step, a multivariable generalised linear model (GLM) using the “glm” function for binomial distribution was constructed using the same outcome variable but with 18 explanatory covariates. This last include human and animal densities (5 variables), trap location, resting habitat, domain, seasons (dry or wet), wind speed, NDVI and NDWI, built-up surfaces per fokontany, ITNs use-to-access ratio, and also also temperature, precipitation and humidity lag yielding the univariate model with the lowest AICc and one type of house (Ravinala-made/non-Ravinala-made, brick-made/non-brick-made, mud-made/non-mud-made, all type of house) yielding the univariate model with the lowest AICc. The Variance Inflation Factor (VIF) of each covariate was checked by using the “check_collinearity” function from the performance package. After excluding covariates with the VIF values greater than 10, the “dredge” function from R MuMIn package^41^ was run to output all possible combinations of covariates. This function displays the best fit model with the smallest AIC. The performance of these best fit model for predicting the presence/absence of given blood are measured using the evaluation metric root mean squared error (RMSE)^42^. RMSE close to zero means that the model’s predictions are very close to the actual values, with a low average error. The final model was used to calculate the Odds ratio (OR) of variables associated to the presence/absence of blood indices of the different hosts for two most abundant mosquito species.

## Results

### Engorged females collected

In total, 1,703 blood-fed females of ten species involved in RVFV and WNV transmission in the literature^25,26^ were collected (Table 2). *Cx. antennatus* (53.36%) and *Cx. quinquefasciatus* (29.13%) were species with the greatest number of blood-fed females. No blood-fed *Cx. antennatus* females were collected from the four most southernly collection sites of the southern domain (S22, S23, S24 and S25). A total of 1,177/1,703 blood-fed females (68.7%) was used for blood meal source identification (Table 2). No blood-fed females were collected in S11.

**Table 2:** Number of blood-fed females (N) collected from the 25 sites with indoor and outdoor collection methods combined, number of females tested for blood meal source identification (n) and 90% of margin of Error (MoE) per species and per collection site.

Sites	An. coustani			An. maculipalpis			An. pauliani			An. squamosus			Cx. antennatus			Cx. decens			Cx. quinquefasciatus			Cx. tritaeniorhynchus			Cx. univittatus			Ma. uniformis			Total	
	*N*	*n*	MoE	*N*	*n*	MoE	*N*	*n*	MoE	*N*	*n*	MoE	*N*	*n*	MoE	*N*	*n*	MoE	*N*	*n*	MoE	*N*	*n*	MoE	*N*	*n*	MoE	*N*	*n*	MoE	*N*	*n*
S01				3	3	–							2	2	–				12	9	12.4				4	2	47.5	1	1	–	22	17
S02	1	1	–	12	5	28.9													2	1	82.3							3	3	–	18	10
S03	1	1	–										24	9	21.4	4	3	23.7	24	15	12.9	6	4	24.5	14	11	9.8	5	3	32.9	78	46
S04													132	110	2.4				209	145	3.5	37	36	0.7				3	2	38.8	381	293
S05				3	3	–	2	2	–				1	1	–																6	6
S06	4	4	–							20	7	24.5	50	38	5.6				3	3	-				7	1	57.6	8	6	15.5	92	59
S07	3	3	–										160	142	1.5				51	48	1.4	33	9	21.2	5	5	71.2	4	1	71.2	256	208
S08	4	3	23.7							1	1	–	72	63	2.4				10	4	32.9							1	1	–	88	72
S09										3	1	77.6	74	53	5.5	4	4	–	2	2	–	4	2	47.5				3	2	38.8	90	64
S10	2	2	–							1	1	–	104	94	1.6				8	6	15.5	3	3	–							118	106
S11																															0	0
S12				23	11	18.3							8	1	54.4				24	7	24.3										55	19
S13																			7	1	57.6										7	1
S14													59	51	2.9				12	6	24.8										71	57
S15													109	22	12.6				36	24	9.3				1	1	–				146	47
S16													4	1	71.2				6	6	–	2	2	-	1	1	–				13	10
S17	1	1	–										19	2	34.7				1	1	–				1	1	–	3	2	38.8	25	7
S18													23	22	1.5				35	3	25.8							2	2	–	60	27
S19	3	2	38.8							1	1	–	28	28	–				16	3	34.5	3	3	38.8	4	1	71.2	1	1	–	56	39
S20	10	3	38.4							1	1	–	34	22	10.1				17	17	–	3	2	–	4	4	–	2	1	82.3	71	50
S21	2	1	82.3							1	1	–	11	11	–				4	4	–										18	17
S22																			2	2	–										2	2
S23																			4	3	23.7										4	3
S24																			12	4	33.1	1	1	–							13	5
S25				11	10	4.73													2	2	–										13	12
	31	21	9.7	52	32	8.9	2	2	-	28	13	17.0	914	672	1.4	8	7	7.8	499	316	2.7	92	62	5.6	41	27	8.9	36	25	8.5	1703	1177

### Blood meal identification

Among the 1,177 blood-fed females tested, DNA from 110 (9,3%) failed (PCR-negative) to identify the blood meal source and 1067 are PCR-positive.

**Fig. 3:**
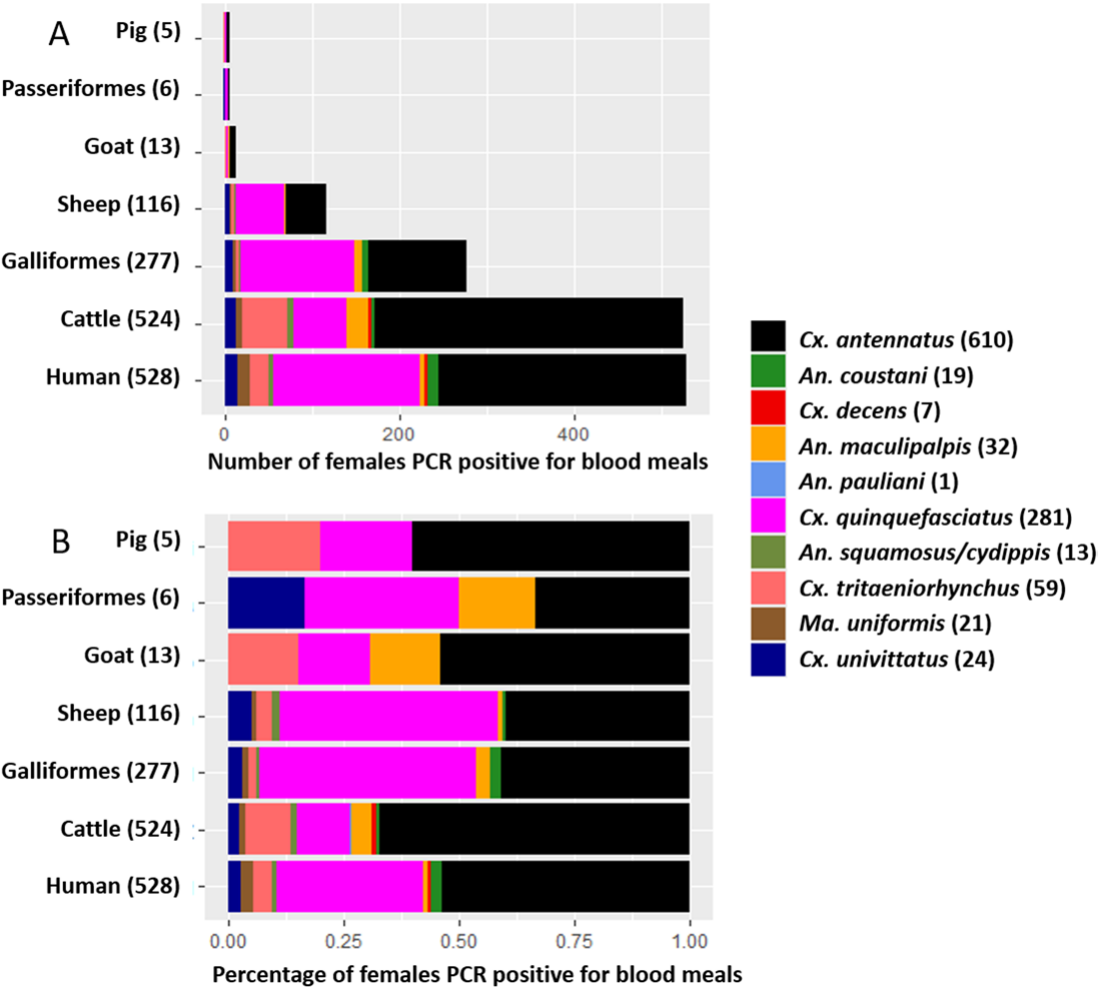
Total number (**A**) and percentage (**B**) of mosquito species in which blood meal sources were identified. Pi : pigs, Ca : cattle, Go: Goat, Hu: Human, Pa: Passeriformes, Ga: Galliformes, Sh: sheep. The number indicated in parentheses after the host species corresponds to the number of female mosquitoes for which this blood meal source was identified. The number indicated in parentheses after the mosquito species name corresponds to the number of mosquitoes for which a blood meal source was identified.

When aggregating the results from single and mixed-blood meals, human and cattle were the dominant vertebrate hosts, with identical proportions: 528 blood-fed females (44.86%) for humans and 524 (44.52%) for cattle and Passeriformes (6 blood-fed females, 0.51%) and pigs (5 blood-fed females, 0.42%) were rarely detected. The species with the greatest number of blood meals identified were *Cx. antennatus* (610/672) and *Cx. quinquefasciatus* (281/316) (Fig. 3).

**Fig. 4:**
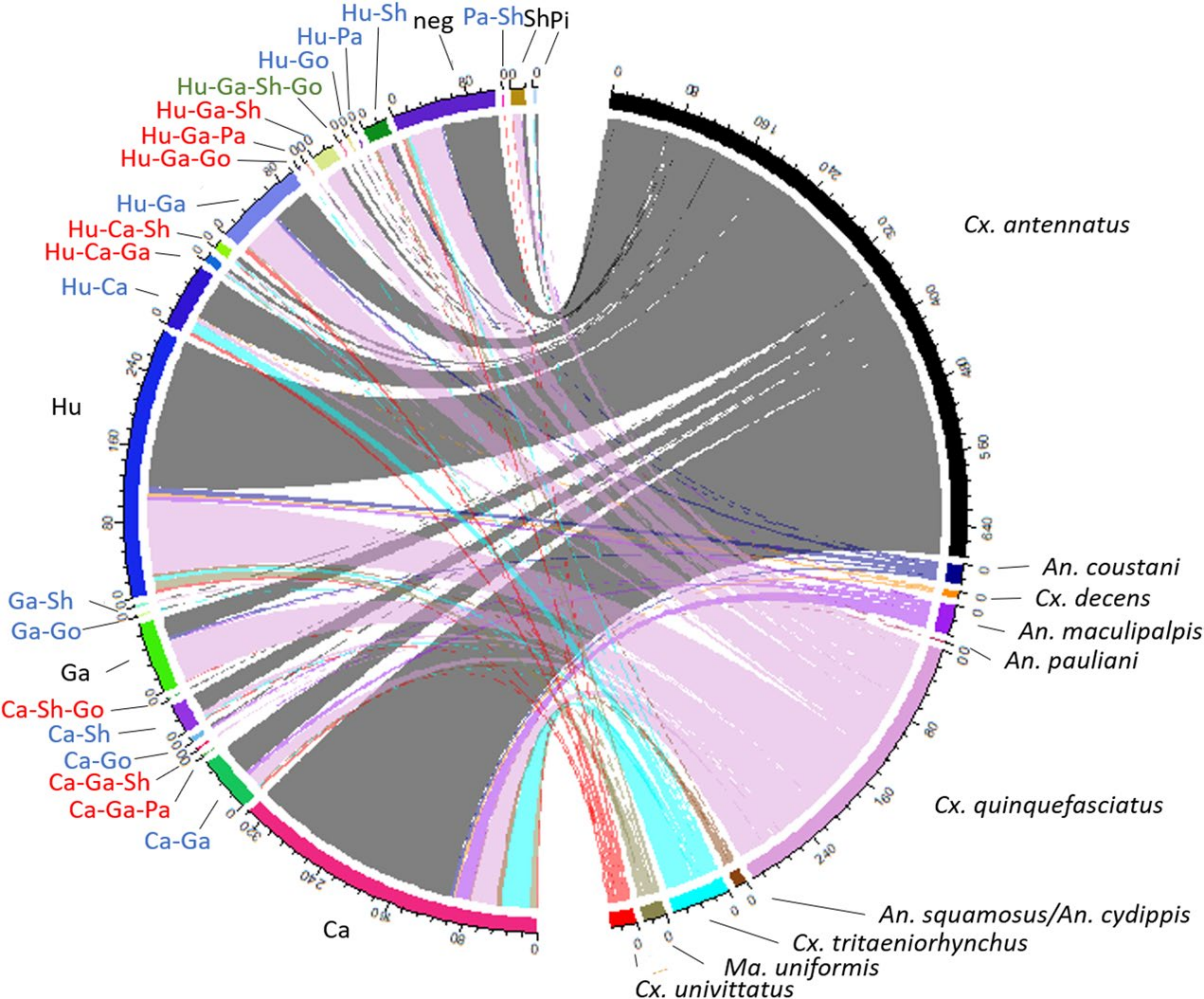
Chord diagram showing the different types of single and mixed blood meals found in tested engorged females of ten mosquito species. Pi: pigs, Ca: cattle, Go: Goat, Hu: Human, Pa: Passeriformes, Ga: Galliformes, Sh: sheep are the blood meals sources. Text in black on the left of the figure corresponds to single blood meal source. Text in blue corresponds to the mixture of two blood sources, in red mixture of three blood sources and in green mixture of four blood sources.

A total of 718 blood-fed females (67.38%) had a single blood meal taken on cattle (*n* = 336, 28.55% of tested blood-fed females), human (*n* = 284, 24.13%), Galliformes (*n* = 77, 6.54%), sheep (*n* = 16, 1.36%) and pig (*n* = 5, 0.42%). Twenty types of mixed blood sources were identified within 349 females. Mixed human and Galliformes blood sources were the most frequent combination observed (*n* = 96, 8.16% of tested blood-fed females, 27.51% of mixed blood sources) (Fig. 4). For *Cx. antennatus* and *Cx. quinquefasciatus*, mixed blood meals accounted for 26.19% (176/672) and 35.44% (112/316) of blood fed females tested respectively (Fig. 4).

When combining goat and sheep as SRu (SRu) and Galliformes and Passeriformes as bird (Bi), the chi-square test revealed statistically significant differences in the distribution of blood meals type in the various species (X-squared = 185.09, df = 27, p-value < 2.2e-16).

**Fig. 5:**
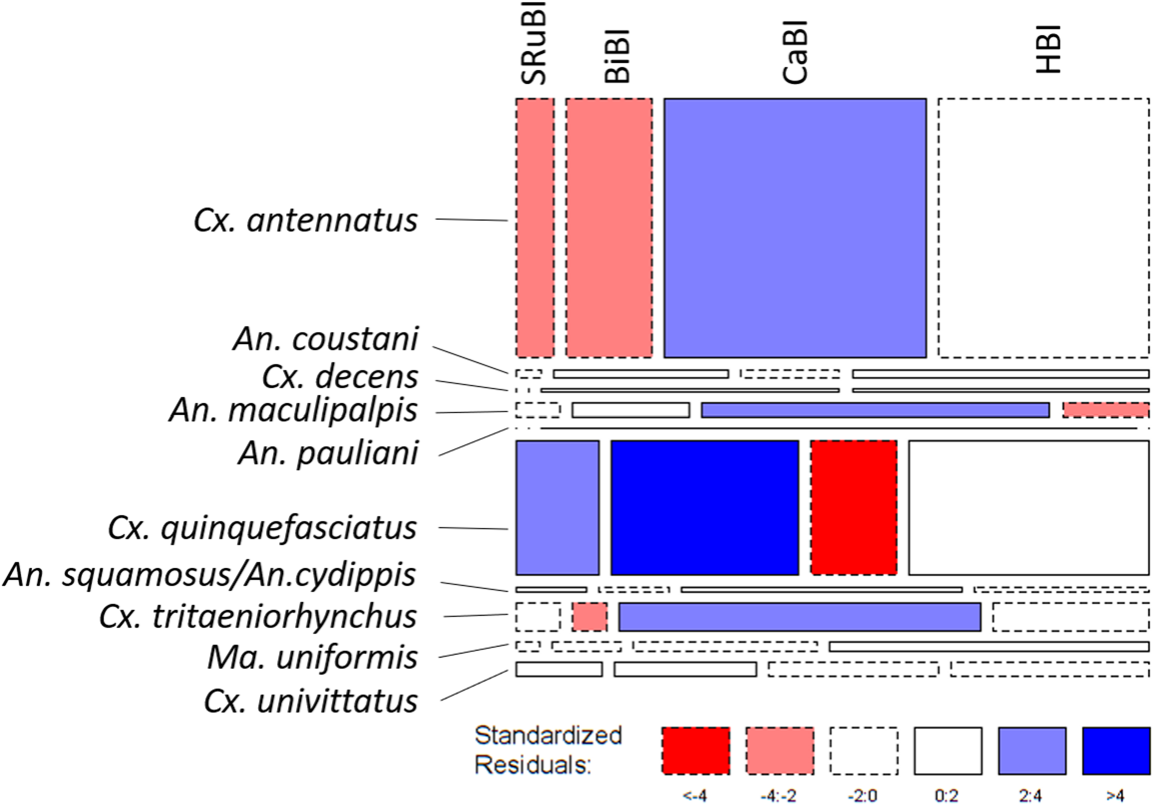
Mosaic plot of human (HBI), cattle (CaBI), bird (BiBI) and small ruminant (SRuBI) blood indices distribution in the ten tested species. Blue indicates a higher value than expected and red indicates a lower value than expected. The size of each cell is proportional to the frequency of elements in the contingency table.

According to the Pearson residuals of the test (Fig. 5), the SRu (SRuBI) and bird (BiBI) blood indices were less associated with *Cx. antennatus* (−3.18 < r_i_ < −2.20) while cattle blood index (CaBI) was more associated with this species (r_i_ = 3.83). Inversely, SRuBI and BiBI were more associated with *Cx. quinquefasciatus* (3.50 < r_i_ < 5.8) while cattle blood index negatively associated with this species (r_i_ = −7.32). CaBI was more associated with *An. maculipalpis* (r_i_ = 2.41) and *Cx. tritaeniorhynchus* (r_i_ = 3.79). Human blood index (HBI) was negatively associated with *An. maculipalpis* (r_i_ = −2.29) while BiBI was negatively associated with *Cx. tritaeniorhynchus* (r_i_ = −2.77). For *Cx. antennatus* and *Cx. quinquefasciatus*, the same results were obtained when the remaining eight species were removed from the analysis.

### Effect of collection methods and trap location

**Fig. 6:**
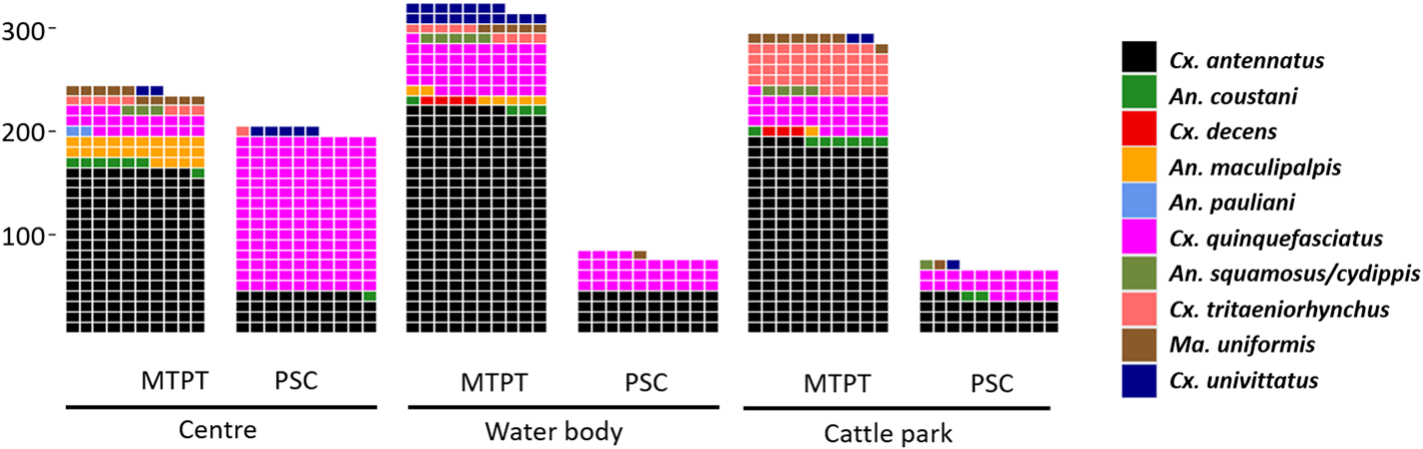
Waffle plots showing the number and distribution of engorged females tested. MTPT: Muirhead Thomson pit traps, PSC: indoor insecticide sprays catch.

Out of a total of 843 tested females collected outdoor using MTPT, the origin of the blood meal source was identified for 770 of them (Fig. 6). The 770 females were composed of 509 *Cx. antennatus*, 96 *Cx. quinquefasciatus*, 59 *Cx. tritaeniorhynchus*, 32 *An. maculipalpis*, 19 *Ma. unformis*, 19 *Cx. univittatus*, 16 *An. coustani*, 12 *An. squamosus/An. cydippis*, 7 *Cx. decens* and one *An. pauliani*. When using PSCs, blood meals were tested for 334 females and the origin of the blood meal source was identified for 297. Among them, 185 females belonged to *Cx. quinquefasciatus* and 101 to *Cx. antennatus*. The remaining 11 females belonged to *Cx. univittatus* (5), *An. coustani* (3), *Ma. uniformis* (2) and *An. squamosus/An. cydippis* (1). The origin of the blood meal source was identified in 392, 355and 320 from centre of village, sites near water body and sites near cattle park respectively (Fig. 6**).**

**Fig. 7:**
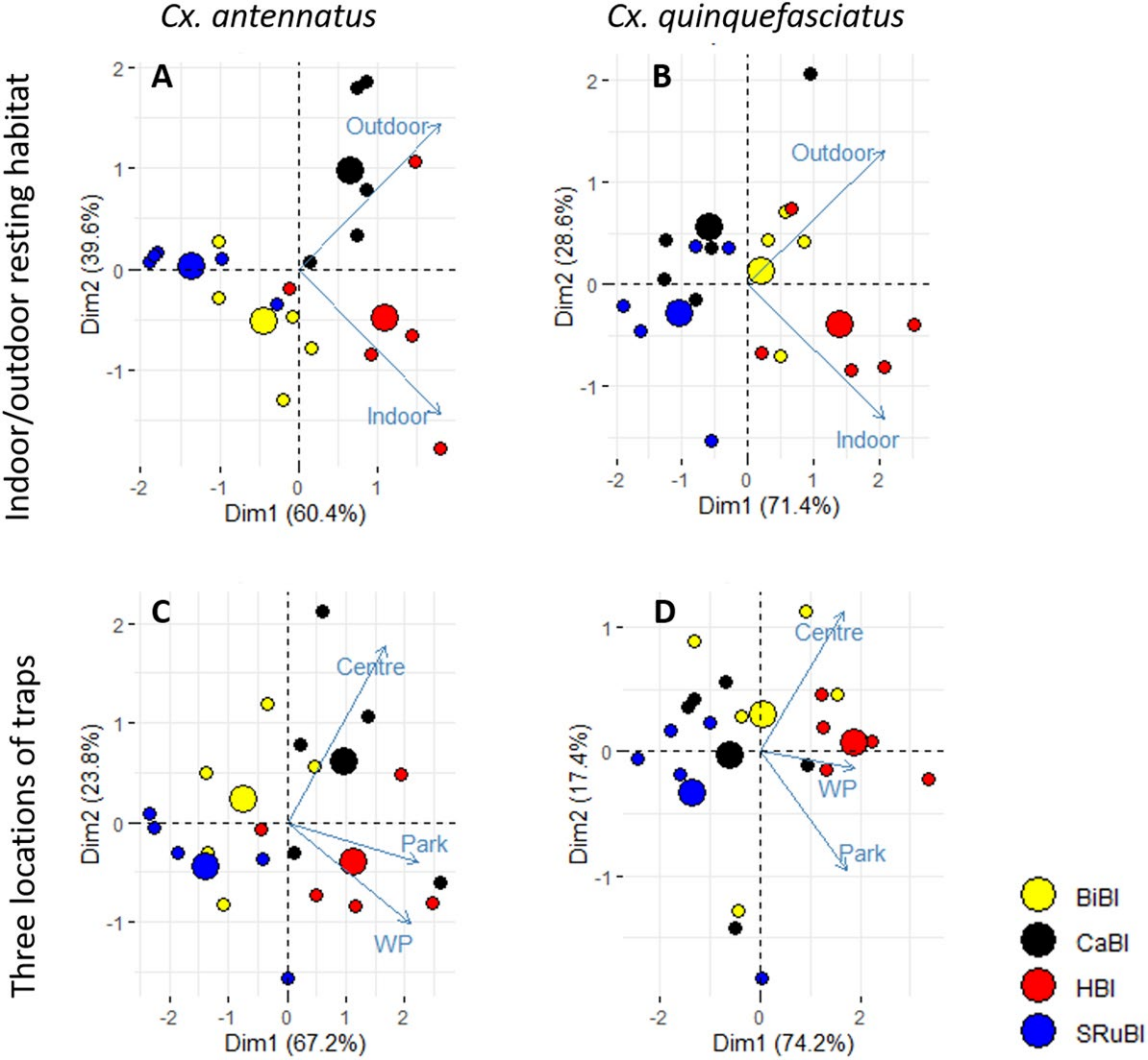
Principal Component Analysis plot showing the distribution of human (HBI), cattle (CaBI), bird (BiBI) and small ruminant (SRuBI) bloods indices between the indoor and outdoor resting sites of *Cx. antennatus* (**B**) and *Cx. quinquefasciatus* (**C**), and between the three trap locations (WB: near water body, Park: near cattle park and Centre: free of cattle park and far of WP) of *Cx. antennatus* (**D**) and *Cx. quinquefasciatus* (**E**).

According to the PCA plot (Fig. 7), indoor collection was negatively associated with CaBI and positively associated with HBI of both species (Fig. 7A and B). Indoor collection of *Cx. antennatus* was also positively associated to a lesser extent with BiBI (Fig. 7A). For *Cx. quinquefasciatus*, indoor collection was negatively associated CaBI and SRuBI was negatively associated with outdoor collection (Fig. 7B). Cattle park and water body were more associated with HBI of *Cx. antennatus* (Fig. 7C). Cattle park was negatively associated with CaBI and water body was significantly associated with HBI for *Cx. quinquefasciatus* (Fig. 7D). SRuBI and CaBI of *Cx. quinquefasciatus* was also negatively associated with cattle park (Fig. 7D).

According to the national scale characterization of *Cx. antennatus* and *Cx. quinquefasciatus* (supplementary Figure S1), the frequency of detecting human, cattle, bird and SRu bloods in engorged females varied among the sites for *Cx. antennatus* (X-squared = 267.74, df = 54, p-value < 2.2e-16) and *Cx. quinquefasciatus* (X-squared = 120.38, df = 66, p-value = 4.997e-05).

### Drivers of blood index

From the univariable analysis (supplementary Table S2), eighteen explanatory variables per type of blood were used in the basic multivariable model. Among these eighteen variables, basic multivariable model includes also one lag period of each of climatic factor (temperature, RH and precipitation), type of house on the basis of smallest AIC (supplementary Table S2). Covariates with strong collinearity (VIF > 10) (supplementary Table S3) were excluded from the subsequent models.

**Fig. 8:**
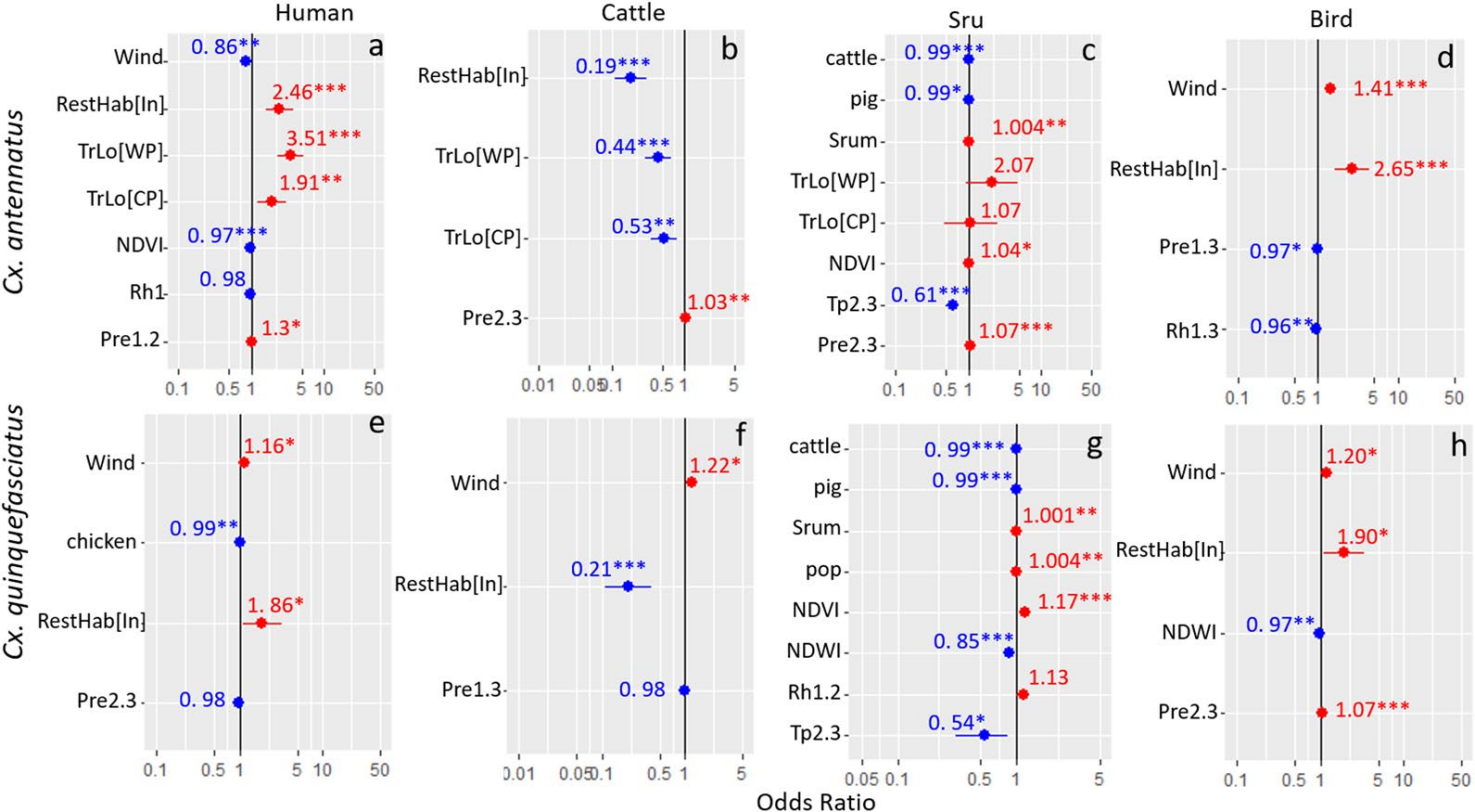
Effects of variables retained in the eight final models on the presence of Human (**a**, **e**), Cattle (**b**, **f**), Small ruminant (**c**, **g**) and Bird (**d**, **h**) blood meals for *Cx. antennatus* (**a**, **b**, **c**, **d**) and Cx. quinquefasciatus (**e**, **f**,** g**, **h**), with 95% confidence interval. In red are the factors with an odds ratio above 1, which increase the risk of presence of a given type of blood meal. In blue are the factors with an odds ratio under 1, which decrease the risk of presence of a given type of blood meal. Therefore, for a given host species, factors in red increase their risk of being bitten by a mosquito whereas factors in blue decrease this risk. RestHab[In]: Indoor mosquito shelter (resting habitat), TrLp: trap loction near water point [WP], and near cattle park [CP], Rh: relative humidity (with 1 to 3 days lag periods); Pre: precipitation (with 1 to 3 days lag periods); Tp: temperature (with 1 to 3 days lag periods). P-values < 0.05 are indicated with *, p-values < 0.01 with ** and p-values < 0.001with ***.

After running the “dredge” function on the subsequent models, eight best fit for presence/absence of human, cattle, bird and SRu blood meals for *Cx. antennatus* and *Cx. quinquefasciatus* were obtained (Table 2). Only presence model of SRu blood did not retained resting habitat (inside *versus* outside) for both species. Among the eight models, only presence model of Human, Cattle and SRu blood of *Cx. antennatus* retained trap location. Wind speed was retained in presence model of human and bird blood for both species and in presence model of cattle blood for *Cx. quinquefasciatus*. The presence model of SRu blood of both species retained NDVI. This last was also retained in the presence model of human blood for *Cx. antennatus*. RH was retained in the presence model of human and bird blood for *Cx. antennatus* and in presence of SRu blood for *Cx. quinquefasciatus*. Only the presence model of bird and SRu blood for *Cx. quinquefasciatus* retained the population size and NDWI. Only presence model of SRu blood for *Cx. quinquefasciatus* did not retained precipitation. Temperature, number of cattle, pigs and small ruminant was only retained SRu models of both species. Number of chickens was only retained in the presence model of human blood meal for *Cx. quinquefasciatus*. RMSE value shown in Table 3 are close to zero (> 0.5), indicating that the eight models are high-performing models to predict the presence/absence of given blood. The odds ratios associated with all retained variables for each fitted model are displayed in Fig. 8.

**Table 3:** Final best fit models of blood meal presence for *Cx. antennatus* and *Cx. quinquefasciatus*. df: degree of freedom, AICc: corrected Akaike information criterion, RMSE: Rroot mean squared error.

Type of blood meal source	Retained variables	df	logLik	AICc	delta	weight	RMSE
*Cx. antennatus*							
Hu	wind + resting habitat + trap location + NDVI + Rh1 + Pre d1.2	8	−421.120	858.5	0.00	0.448	0.466
Ca	resting habitat + trap location + Pre d2.3	5	−424.901	859.9	0.00	0.181	0.468
Bi	wind + resting habitat + Pre1.3 + Rh1.3	5	−261.216	532.5	0.00	0.565	0.346
Sru	cattle + pig + Sruminant + trap location + NDVI + Tp d2.3 + Pre d2.3	9	−141.938	302.1	0.00	0.043	0.245
*Cx. quinquefasciatus*							
Hu	wind + chicken + resting habitat + Pre d2.3	5	−208.238	426.7	0.00	0.026	0.483
Ca	wind + resting habitat + Pre d1.3	4	−133.231	274.6	0.00	0.230	0.369
Bi	wind + resting habitat + NDWI + Pre d2.3	5	−197.047	404.3	0.00	0.357	0.466
Sru	cattle + pig + Sruminant + pop + NDVI + NDWI + Rh d1.2 + Tp d2.3	9	−115.586	249.8	0.00	0.077	0.342

For human (Fig. 8a), cattle (Fig. 8b) and SRu (Fig. 8c), increase of precipitation increased exposure risk to *Cx. antennatus* bites (1.03 < OR < 1.3, *p* < 0.05). Opposite, increased precipitation decreased the risk of exposure of birds (Fig. 8d) to *Cx. antennatus* OR = 0.96, *p* < 0.05). Increase of precipitation has no effect on the exposure of human (Fig. 8e) and cattle (Fig. 8f) to *Cx. quinquefasciatus* bite. For *Cx. quinquefasciatus*, if the presence model of SRu (Fig. 8g) blood did not retained precipitation, the increase of precipitation increased exposure risk of birds (Fig. 8h) to bites of this species (OR = 1.07, *p* < 0.001). SRus were protected against *Cx. antennatus* and *Cx. quinquefasciatus* bites when the temperature increased (0.5 < OR < 0.65, *p* < 0.05). Birds were also protected against *Cx. antennatus* bites when the RH increased (OR = 0.96, *p* < 0.001). Increase of wind speed appeared to protect humans from being bitten by *Cx. antennatus* (OR = 0.86, *p* < 0.01). Birds were more exposed to *Cx. antennatus* bites when wind speed increased (OR = 1.41, *p* < 0.001). For *Cx. quinquefasciatus*, increase of wind speed was the risk of being bitten for humans (OR = 1.16, *p* < 0.05), cattle (OR = 1.22, *p* < 0.05) and birds (OR = 1. 20, *p* < 0.05). Indoor collections increased the chance to obtain *Cx. antennatus* and *Cx. quinquefasciatus* with human (1.8 < OR < 2.50, *p* < 0.05) and bird blood (1.2 < OR < 1.5, *p* < 0.001) and inversely decreased the chance to obtain *Cx. quinquefasciatus* with cattle blood (OR = 0.21, *p* < 0.001). Being located near a cattle park and a water body were associated with a 2- to 3-fold increase in the risk for humans of being bitten by *Cx. antennatus* (1.9 < OR < 4, *p* < 0.01), and constituted a protective factor for cattle against *Cx. antennatus* bites (0.4 < OR < 0.55, *p* < 0.01). Increase in NDVI-value (OR = 1.04,*p* < 0.001) increased the risk for SRus (1.00 < OR < 1.20, *p* < 0.01) to be bitten by both mosquito species, and decreased the risk for humans to be bitten by *Cx. antennatus* (OR = 0.97, *p* < 0.001). Increase in NDWI-value was associated a decreased risk for birds and SRus (0.8 < OR < 0.98, *p* < 0.01) to be exposed to *Cx. quinquefasciatus* bites. The increase in number of chickens was a protective factor for humans (OR = 0.99, *p* < 0.01) against *Cx. quinquefasciatus* bites. The increase in number of cattle was a protective factor for SRus (OR = 0.99, *p* < 0.01) against *Cx. antennatus* and *Cx. quinquefasciatus* bites. Increase in population density constituted a risk factor for SRus to be bitten by *Cx. quinquefasciatus* (OR = 1.004, *p* < 0.01). Increase in number of SRus was a risk factor for SRus to be bitten by both species (1.00 < OR < 1.005, *p* < 0.01).

## Discussion

In Madagascar, numerous data on the feeding preferences of mosquito vectors of RVFV and WNV^10,21–23^ are limited in time and space and does not allow to evaluate feeding variations among populations and species. Here, this study displays the first wide-scale study targeting feeding behaviour of mosquito species associated with both arboviruses in Madagascar.

The ten analysed species include *Cx quinquefasciatus*,* Cx. univittatus*,* Ma. uniformis*, and *Cx. tritaeniorhynchus*, considered as major vector species, *An. coustani*,* Cx. decens* and *Ae. circumluteolus* as candidate vector species and *An. maculipalpis* and *An. pauliani* as potential vector species of WNV^25,26^ as well as *Cx. antennatus*, considered as major vector species and *An. squamosus*,* An. coustani*,* Cx. decens*,* Cx. univittatus*,* Cx. quinquefasciatus*,* Cx. tritaeniorhynchus*, and *Ma. uniformis* as candidate vector species of RVFV^25^.

Trophic preference was variable both across periods and sites of mosquito collections, and differed according to the resting habitat (indoor/outdoor) or collection method (PSC/MTPT) used. The limited species diversity (*n* = 10) analysed in our survey is probably linked to the choice of the collection methods (MTPT and PSC), which provide fewer number of mosquito species than other traps such as light traps^43^ or QUEST (Quadrant Enabled Screen Trap) methods^22^. These methods are the most adequate to investigate mosquito trophic preference^21^, since they specifically target individuals which have taken a blood meal and since other methods targeting these individuals such as host landing captures are time consuming and raise ethical issues.

Using PCR, the blood meal source was identified from more than 90% of females tested. PCR-based methods are fast and have great sensitivity^29^. Blood meal source could not be identified for 110 (9.34%) blood-fed females suggesting the occurrence of other blood meal sources not tested or the non-amplification of DNA due to low DNA quantity or DNA degradation because of the digestion process^44^. No horse-specific primers^8^ was used because no horses were present in any of our study sites.

In Madagascar, this is the first report for *Cx. antennatus*,* Cx. quinquefasciatus*,* Cx. tritaeniorhynchus* and *Ma. uniformis* feeding on pigs. These mosquito species have already been reported to feed on pig in other countries^45–47^. Our study is the first to report the detection of passerine blood from *Cx. quinquefasciatus* and *Ma. uniformis*, which were already reported to feed on Passeriformes in multiple occasions in other countries^48,49^.

Most mosquito blood meals were taken from humans and cattle, probably due to high number of humans and cattle, combined with their relatively large size and thus CO_2_ emitting capacity, which is a proxy of their attractivity^50^.

It would be ideal to model the variation of the blood index in time and space as reported for *Aedes* mosquitoes^3^. Modeling the presence of blood in a wild female would be much more informative than modeling mosquito blood index due to the fact that blood meal analysis is a proxy for host preference^51^.

Among ten mosquito species analysed, 67.38% of the tested engorged females had taken a single blood meal and 29.65% mixed blood meals. This result highlights the importance of opportunistic feeding behaviour of all these species as already reported in other studies^25,26^. Their opportunistic feeding behaviour increases the probability that they serve as bridge vectors between infected birds and humans^52^ and between infected ruminant and humans^53^, and in consequence constitute an important risk for WNV and RVFV transmission^25,26^. However, changes in the gut microbiota in response to mixed blood meals have the potential to alter vector susceptibility to pathogens/parasites^54^, but this has not been proven for RVFV and WNV.

Models were developed for the two species with the greatest number of engorged females: *Cx. antennatus* (672 engorged females) and *Cx. quinquefasciatus* (316 engorged females). Our study revealed for the first time that presence/absence of blood meal of a given host origin is dynamic and driven at least by the wind speed, trap location in villages, mosquito resting sites, environmental factors, climatic factors and number of available vertebrate hosts. Surprisingly, the association between an increase in the number of a given host with the presence of blood meals from that host was only observed for SRu. For *Cx. quinquefasciatus*, there is a cross-association between the presence of SRu blood with increase in number of human (Fig. 8g). These results confirm that mosquito trophic preference is readily overruled by the physical abundance of available hosts^4^ and potentially depending on endophilic/exophilic and endophagic/exophagic behaviours of this species (not tested in this study).

The negative effects of the number of chickens, cattles and pigs on the exposure of human (Fig. 8e) and SRus (Fig. 8c, g) to bites of *Cx. quinquefasciatus* and *Cx. antennatus* recall the dilution effect^55^, despite their low OR (close to 1). This dilution effect occurs if greater host diversity leads to lower contact rates between vectors and susceptible hosts^55^.

In this study, the endophilic mosquito populations of *Cx. antennatus* and *Cx. quinquefasciatus* were expected to have fed on human blood. Indeed, indoor resting females are more likely to have fed on humans^4^ and mosquitoes resting outdoor to exhibit a high level of ruminant blood meals^56^. Similarly, cattle were highly exposed to exophilic mosquitoes. Birds are highly exposed to the endophilic mosquitoes of both species suggesting that endophilic mosquito readily bites not only human but also domestic birds that are placed indoor. However, the result of this study does not to analyze the behaviour of an exophagous but endophilic mosquitoes.

The analysis also shows that NDVI is a protective factor for humans against *Cx. antennatus* bites. This results probably arise from the fact that increase in terrestrial plants might reduce contact between this species and human, by providing favourable outdoor resting places for exophilic mosquito population^57^. In this study, NDWI is protective factor for domestic animals against *Cx. quinquefasciatus* bites. NDWI has been linked to mosquito density as it can be a proxy of breeding habitat^58^.

Our study also reports that trap location close to water body is a risk factor for humans to be bitten by *Cx. antennatus*. This result could suggest that the proximity of dwellings to breeding sites, increased human population exposure to mosquito bites^59^.

Mosquitoes fly actively, searching for opposite sex to mate, host, resting site and oviposition site in the absence of wind or if the wind speed is less than 2 m/s^60^. It is demonstrated that an increase in wind speed lead to a decrease in the total biting rate of exophagic mosquito populations^61^.

Wind also decreases mosquito collections by diluting mosquito attractants^62^. Here, our study report that increase in wind speed is a protective factor for human against *Cx. antennatus* bites, but constitutes a risk factor for human for *Cx. quinquefasciatus* bites.

This result could be due to the fact that *Cx. antennatus* is more exophilic and exophagic than *Cx. quinquefasciatus*^63^. Increase in wind speed could reduce exophilic *Cx. antennatus* entry into houses or prevent endophilic *Cx. quinquefasciatus* from exiting, by countering upwind progress^62^.

Exposure of humans to at least *Cx. antennatus* bites was expected higher than cattle exposure to bites of mosquitoes near cattle parks (Fig. 8a, b). The presence of ruminants in and near human habitations attracts mosquitoes, which may increase their risk of transmitting vector-borne pathogens to humans. The proximity of cattle to human dwellings diverts host-seeking mosquitoes^64^.

The low exposure of humans to *Cx. quinquefasciatus* bites when the number of chicken increase recall the zooprophylaxy effect which is a decrease in the human biting rate in favour of mosquitoes feeding on the surrounding domestic animals^65^. In Madagascar, the success of zooprophylaxy depends on mosquito vector species and their host feeding preferences^66^, and probably to the certain types of animals that exert stronger zooprophylactic effects than others^67^. These high exposition of humans to *Cx. antennatus* bites near cattle park support the fact that zooprophylaxy measures remain controversial for African countries when considering arboviruses^68^. Considering that both species are opportunistic feeders^8^, they could modify their host use according to variations in host availability across habitats and seasons^4^. With *Culex quinquefasciatus*, it has been shown that chicken exert stronger zooprophylactic effects than the remaining vertebrate hosts.

Finally, even though season was not retained in the final fitted model, mosquitoes may express different host preferences depending on the season^4^. In this study, the season effect might result from the effect of temperature, humidity and precipitation on mosquito behaviour, not from migratory or ruminant bird events^4^.

Precipitation has been shown to increase mosquito attraction to skin odor baits^69^ which could explain significant exposure of human, cattle, and SRu to *Cx. antennatus* bites. At the same time, precipitation increase also the creation of larval breeding sites^70^, favouring mosquito abundance.

Temperature is known to impact biting rate: increasing temperatures increase biting rate until a given threshold above which the biting rate declines^71^, supporting the fact that increase in temperature is a protective factor for SRu against *Cx. antennatus* bites. These results confirm that RVFV and WNV transmission involves mainly exophilic and zoophilic species^25,26^. The use of indoor residual spraying and insecticide treated nets are unlikely to have a substantial impact on RVFV and WNV transmission risk in Madagascar^25,26^. These indoor vector control measures only offer protection against endophilic and endophagic species^72^. Opposite, methods targeting larvae or outdoor resting mosquitoes, or ensuring the protection of animals are needed to interrupt mosquito-borne transmission of RVFV. These will need to be implemented alongside to target the reduction of non-vectorial transmission pathways of RVFV.

## Conclusion

Ten mosquito’s species collected all over Madagascar show generalist feeding patterns and may act as bridge vectors for WNV and RVFV. *Culex quinquefasciatus* and *Cx. antennatus* trophic preference is modulated not only by climatic and environmental factors but also by habitat types, resting places and diversity and number of available vertebrate hosts. The variations of trophic behaviour observed may contribute to the heterogeneous incidence of both of WNF and RVF. This study provides an essential step towards a stronger evidence-based determination of risk of arboviruses transmission in humans and livestock. The use of other tools such as larval control or adult mosquito mass trapping to supplement indoor vector control in Madagascar needs to be explored.

## Supplementary Information

Below is the link to the electronic supplementary material.

Supplementary Material 1

Supplementary Material 2

Supplementary Material 3

Supplementary Material 4

## Data Availability

The datasets used and analysed during the current study available from the corresponding author on reasonablerequest.

## Declarations

Figure legenfds.
