# Supplementary figures and images for: Determining factors of the blood feeding behaviour of mosquito vectors of West Nile and Rift Valley fever viruses in Madagascar
Source: Sci Rep. 2026 Apr 28;16:19685. doi: 10.1038/s41598-026-46448-3 (PMC13315263; doi:10.1038/s41598-026-46448-3)

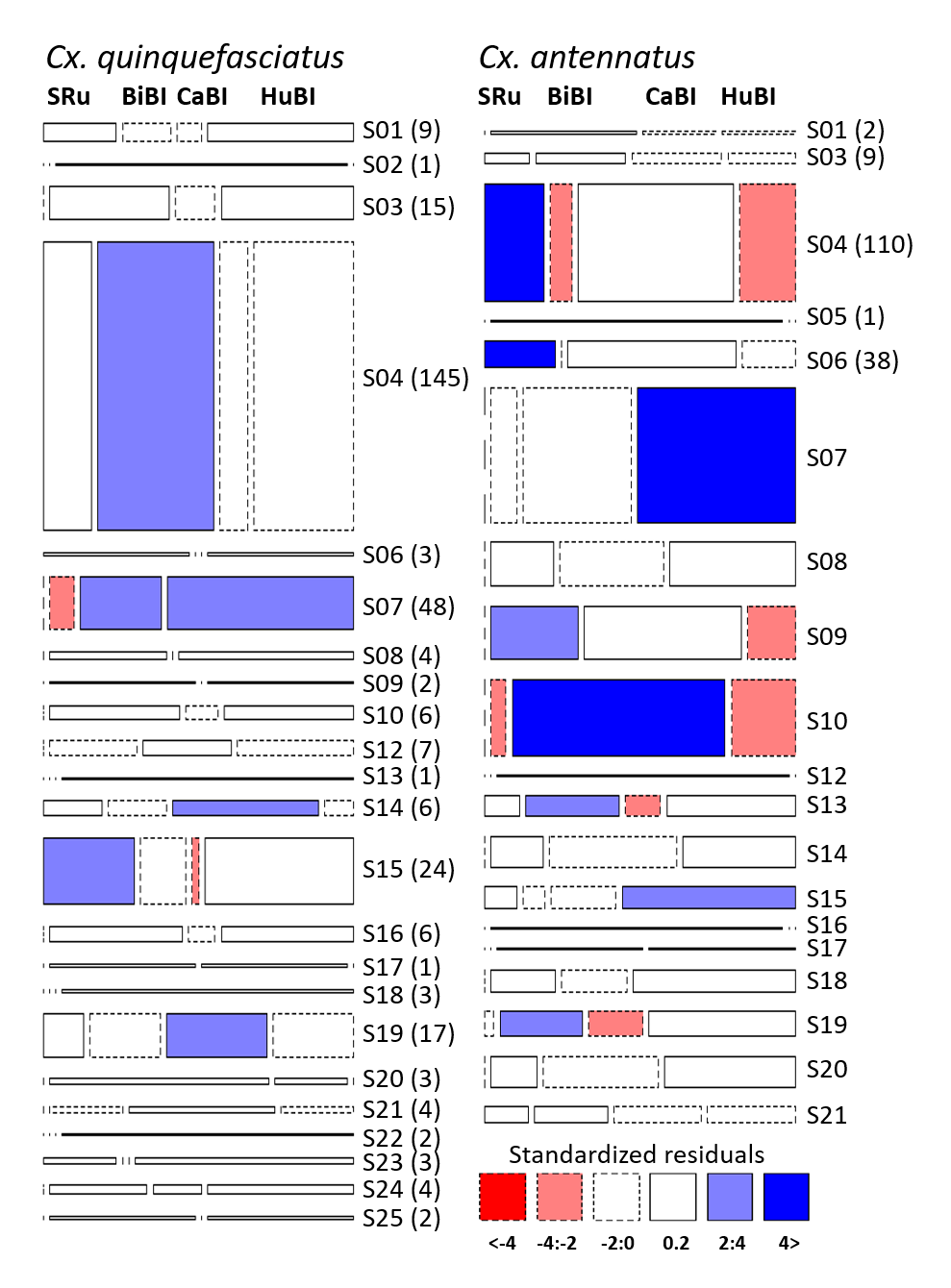

Supplement: Supplementary file 4 — Supplementary Material 4 [file 41598_2026_46448_MOESM4_ESM.tiff]
